# A Comprehensive Commentary on the Multilocular Cystic Renal Neoplasm of Low Malignant Potential: A Urologist’s Perspective

**DOI:** 10.3390/cancers14030831

**Published:** 2022-02-06

**Authors:** Tomas Pitra, Kristyna Pivovarcikova, Reza Alaghehbandan, Adriena Bartos Vesela, Radek Tupy, Milan Hora, Ondrej Hes

**Affiliations:** 1 Department of Urology, Faculty Hospital in Pilsen, Charles University in Prague, 30599 Pilsen, Czech Republic; veselaad@fnplzen.cz (A.B.V.); horam@fnplzen.cz (M.H.); 2 Sikl’s Department of Pathology, Faculty Hospital in Pilsen, Charles University in Prague, 30599 Pilsen, Czech Republic; pivovarcikovak@fnplzen.cz (K.P.); hes@fnplzen.cz (O.H.); 3 Department of Pathology, Faculty of Medicine, University of British Columbia, Royal Columbian Hospital, Vancouver, BC V6T 1Z4, Canada; reza.alagh@gmail.com; 4 Department of Radiology, Faculty Hospital in Pilsen, Charles University in Prague, 30599 Pilsen, Czech Republic; tupyr@fnplzen.cz

**Keywords:** kidney, cystic tumor, imaging, magnetic resonance, surgery

## Abstract

**Simple Summary:**

Multilocular cystic renal neoplasm of low malignant potential (MCRNLMP) is a cystic renal neoplasm with an excellent prognosis. This neoplasm was previously named as “multilocular cystic renal cell carcinoma”, which is now considered obsolete. In 2016, the WHO distinguished this neoplasm of low malignant potential from cystic renal cell carcinomas, which have some overlapping morphologic features.

**Abstract:**

Multilocular cystic renal neoplasm of low malignant potential (MCRNLMP) is a cystic renal tumor with indolent clinical behavior. In most of cases, it is an incidental finding during the examination of other health issues. The true incidence rate is estimated to be between 1.5% and 4% of all RCCs. These lesions are classified according to the Bosniak classification as Bosniak category III. There is a wide spectrum of diagnostic tools that can be utilized in the identification of this tumor, such as computed tomography (CT), magnetic resonance (MRI) or contrast-enhanced ultrasonography (CEUS). Management choices of these lesions range from conservative approaches, such as clinical follow-up, to surgery. Minimally invasive techniques (i.e., robotic surgery and laparoscopy) are preferred, with an emphasis on nephron sparing surgery, if clinically feasible.

## 1. Introduction

Multilocular cystic renal neoplasm of low malignant potential (MCRNLMP) is a benign cystic lesion of the kidney, which was previously known as multilocular cystic renal cell carcinoma (MCRCC). This entity was initially described in 1982 by Lewis et al. [1]. Over time, the diagnostic criteria have changed from initially being defined as a tumor in which solid typical renal cell carcinoma exhibit less than 10% of the total mass [2]. A subsequent proposal suggested a cutoff point of 25% [3]. Finally, the 2012 International Society of Urological Pathology (ISUP) Vancouver Modification of the 2004 World Health Organization (WHO) Histologic Classification of Kidney Tumors recommended the re-designation of MCRCC as a multilocular cystic renal neoplasm of low malignant potential (MCRNLMP) [4,5]. MCRNLMP has a similar genetic profile and histopathological characteristics to that of clear cell renal cell carcinoma (CCRCC), but with a completely different prognostic feature with no progression or metastatic potential, because there are no reports of disease progression or metastases to date [6,7,8,9,10,11]. The 2016 WHO classification defined MCRNLMP as a tumor entirely composed of multiple cysts, of which the septa contain small groups of clear cells without expansive growth, and is morphologically indistinguishable from low-grade CCRCC [12]. It should be noted that MCRNLMP follows strict histologic criteria that would allow any expansive growth, the presence of which qualifies the tumor as a cystic CCRCC [5,12].

## 2. Clinical Characteristics

MCRNLMP is a relatively rare entity, representing approximately less than 1% of all renal tumors, affecting middle-aged adults with a slight male predominance [2,13,14,15]. Most cases are asymptomatic and found incidentally. However, in the setting of large tumors, patients may present with gross hematuria, flank pain, palpable mass and abdominal discomfort, and sometimes digestive symptoms [3,16].

## 3. Imaging Studies

**Figure 1:**
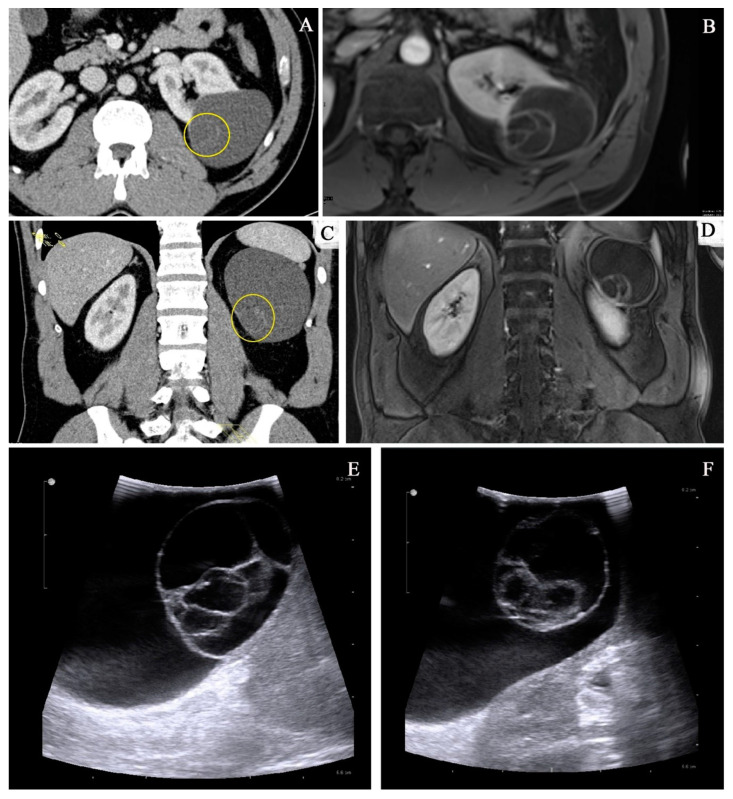
Imaging methods: comparison of CT imaging (**A**,**C**) and MRI (**B**,**D**) of the same lesion. There is a clearly visible benefit of MRI in imaging of the inner architecture with more precise imaging of the septa. (**E**,**F**) Intraoperative ultrasound image of MCRNLMP.

MCRNLMP is often initially identified on B-mode ultrasound as a well-defined multilocular cystic lesion with numerous septa, filled with serous or complicated fluid. Given the cystic nature of the lesion, further investigation by computed tomography (CT) using contrast agent is still the gold standard in classification and subsequent decision making in the field of cystic tumors of the kidney. The Bosniak classification with five groups (I, II, IIF, III and IV) is used as standard for defining cystic tumors of the kidney on CT. Results of CT scans and strict definitions of the Bosniak category of the cystic lesion are crucial for the further management of these lesions [17,18,19,20,21]. According to Bosniak, great parts of MCRNLMPs are defined/described as Bosniak category II, IIF or III [22,23]. In indeterminate cases where the CT imagining shows Bosniak category IIF–III, other imaging modalities (i.e., MRI), with greater precision and better visualization of the inner architecture of the septa, can be utilized [24,25] (Figure 1). In patients who cannot undergo CT or MRI, the preferred modality choice would be contrast-enhanced ultrasound (CEUS) [26,27,28,29,30]. This modality is now recognized as a diagnostic tool with at least the same effectiveness and imaging precision of cystic lesion as contrast-enhanced magnetic resonance or contrast-enhanced computed tomography [21,31,32,33].

### 3.1. Bosniak Classification

The first time the Bosniak classification was proposed and published was in 1986 [18]. In the following years and decades, this classification underwent several updates. Originally, four groups were expanded into five groups, adding a new unit—Bosniak IIF. The latest update of the Bosniak classification came in 2019 [34,35,36].

Each Bosniak group is evaluated according to the structure of the cystic lesion, the number of septa, the thickness and regularity/irregularity of the septa and wall, the presence of contrast enhancement in the septa, and the presence of calcifications or soft-tissue nodules. 

**Bosniak I group**—simple cyst, uncomplicated. Defined by a thin wall, no septa, and no contrast enhancement.

**Bosniak II group**—minimally complex cyst, minimally complicated. Defined by a thin wall and septa, calcifications can be present, and no contrast enhancement.

**Bosniak IIF group**—slightly thickened wall, thin septa with visible, but not measurable enhancement, and the presence of calcifications.

**Bosniak III group**—indeterminate cystic tumor, thickened, irregular wall and septa, and measurable contrast enhancement.

**Bosniak IV group**—cystic tumor, soft-tissue nodules with measurable enhancement.

### 3.2. Differential Diagnostics

Due to its cystic nature, MCRNLMP could be misdiagnosed as another cystic tumor of the kidney, according to imaging studies. In differential diagnostics, it could be diagnosed as a hemorrhagic or inflamed cyst, or mixed epithelial and stromal tumor of the kidney (MESTK) [22]. A recent study from Song et al. [33] described a series of six cases of Xp11 translocation renal cell carcinoma, which have some morphological features mimicking MCRNLMP. Entities in the differential diagnosis are summarized in Table 1.

## 4. Therapeutic Management

The therapeutic management of cystic lesions of the kidney (including MCRNLMP) is still based on the results of imaging studies and precise categorization according to the Bosniak classification system. Each Bosniak category is associated with the individual risk of malignancy and the malignity rate. The malignity rate is based on typical signs of each group-complexity of the lesion and the characteristics mentioned above (Section 3.1). The malignity rates in Bosniak I and II, based on recent cohorts in the literature, are given as 3.2% and 6%, respectively [37]. The Bosniak IIF malignity rate is reported as 6.7% [37] or 18% [38]. The Bosniak III malignity rate is 55.1% [37]. In Bosniak IV, the malignity rate is reported as 91% [37]. 

There is no need for intervention or regular follow-up in Bosniak I and II category, except for large lesions with clinical symptoms. Bosniak IIF is a cystic lesion, where regular follow-up is recommended. However, no strict consensus protocol has been provided, and the follow-up protocols or eventual surgical intervention are still controversial. Follow-up is the preferred choice of management. There are multiple proposed recommendations in the literature on how to manage these lesions. Bosniak et al. proposed a follow-up regimen based on CT scans 6 months after diagnosis. In cases of no progression, another imaging study should be performed once per year [39]. Another study from Weibl et al. suggested follow-up CT scans every 6 months in the first 2 years, and then continuing with the imaging study once every year. The authors incorporated MRI in the follow-up regimen, which should be performed minimally in the first 4 years of follow-up [40,41]. For Bosniak III category lesions, there are two options available: (1) surgical treatment, possibly with minimally invasive nephron sparing surgery with regard to the oncological radicality of the procedure; and (2) strict clinical follow-up, as per the recent guidelines of the European Association of Urology [42]. Bosniak IV is treated as a solid tumor of the kidney, with the surgical interventions described above. 

## 5. Pathological Findings

### 5.1. Macroscopic Findings

**Figure 2:**
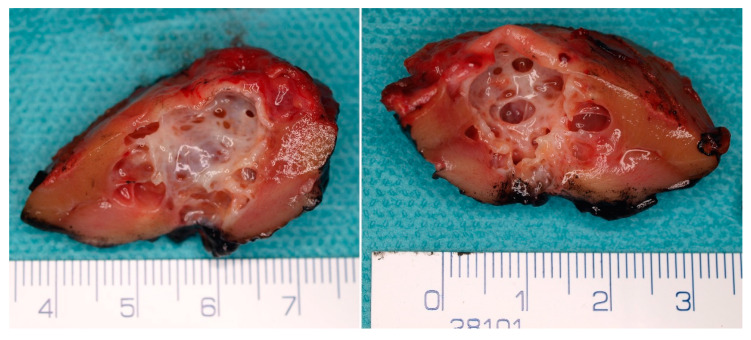
Macroscopic appearance of the MCRNLMP specimen from nephron sparing surgery. There is a multicystic lesion with a thin septa and variable sized cystic spaces without solid expansion.

**Figure 3:**
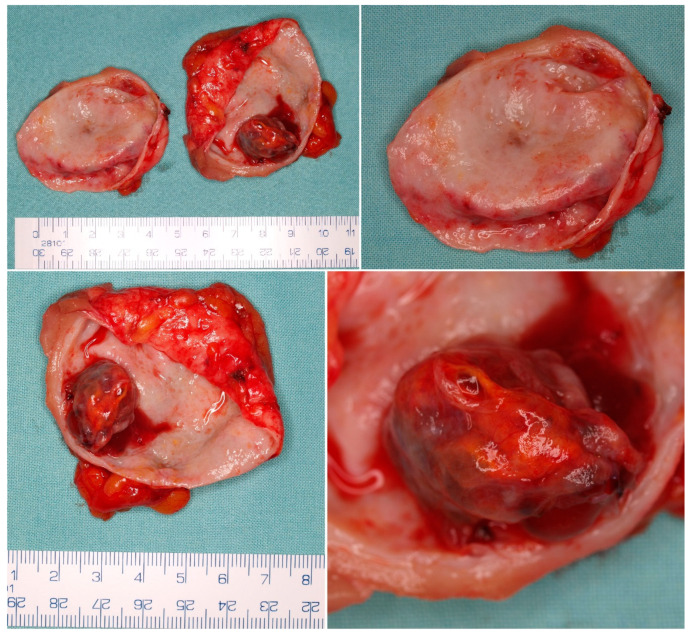
Macroscopic appearance of an MCRNLMP specimen from nephron sparing surgery. The dominant cystic space contains smaller cystic expansion. The absence of solid mass is crucial for the diagnosis of MCRNLMP, and must be proved by microscopic examination of the specimen.

MCRNLMP exclusively consists of variably large non-communicating cysts (0.4–14 cm) [9,10], which are separated by thin septa and filled with serous, gelatinous, hemorrhagic, or mixed fluid (Figure 2 and Figure 3). There are no solid components in these lesions, and, in fact, the presence of such solid nodules would not be compatible with the diagnosis of this entity [9,10,12,43]. Most patients have unilateral lesions with no laterality predominance [3,9,44].

### 5.2. Microscopic Findings

**Figure 4:**
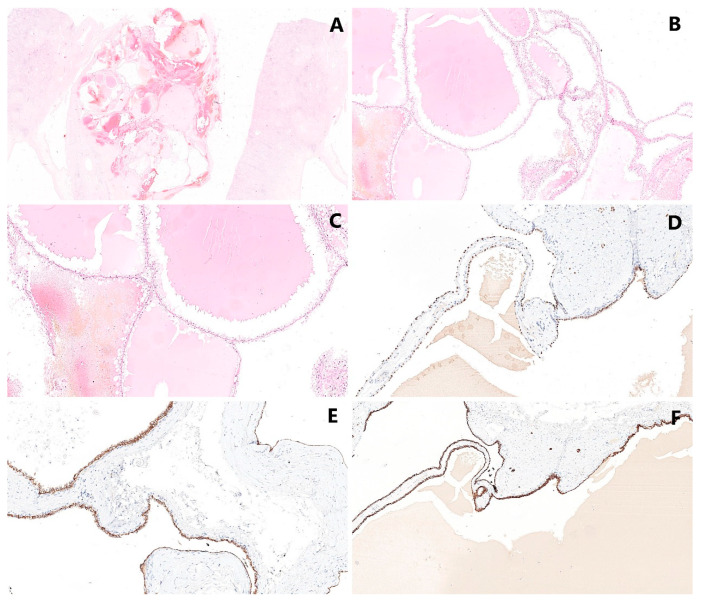
Histological appearance of MCRNLMP: (**A**,**B**) The lesion is characterized by the formation of cystic spaces—various sized cysts are separated by thin, fibrous septa (magnification 10×, resp. 60×). (**C**) The epithelial lining is composed by neoplastic cells with clear cytoplasm arranged in a single layer (magnification 160×). (**D**) The epithelial lining is positive in PAX8 (magnification 10×). (**E**) Equally, carbonic anhydrase IX (CAIX) shows positivity in neoplastic cells (magnification 10×). (**F**) Strong immunoreactivity was proved in CK7 (magnification 10×).

The neoplasm is composed of the cystic spaces lined by clear cells, exhibiting low-grade nuclei without nucleoli (WHO/ISUP grade 1–2). No expansive/solid nodular growth of clear tumor cells, necrosis, vascular invasion or sarcomatoid changes have been noted in MCRNLMP. In rare cases, the linings of cysts may show multilayering, granular cytoplasm of cells and the formation of small intracystic papillae. Furthermore, the septa may exhibit calcification or ossification [12,45] (Figure 4).

### 5.3. Immunohistochemical Findings

Neoplastic cells are typically PAX2-, PAX8-, and carbonic anhydrase IX (CAIX)-positive [46,47,48]. In wider immunohistochemical panels, MCRNLMP is usually negative in α-methylacyl-CoA-racemase, progesterone and estrogen receptor. Strong immunoreactivity was proven in EMA, CAM5.2 and CK7 [44,49].

Some authors used less common immunohistochemical staining techniques in their immunohistochemical studies—Kuroda et al. demonstrated the immunoreactivity of the cytoplasm of tumor cells in adipophilin which corresponded to lipid droplets [44]. Adipophilin expression in CCRCC has previously been reported, which may reflect a close relationship between MCRNLMP and CCRCC [50]. Kim et al. recently examined a number of immunostains between MCRNLMP and CCRCC. According to their study, the expressions of TGAse-2 and Ki-67 were significantly different between these two groups [12,51].

### 5.4. Molecular Genetic Findings

*VHL* gene mutations were found in 25% of MCRNLMP [47], and deletions of chromosome 3p in 74% of cases in comparison with 89% of CCRCC. These findings can support the concept of MCRNLMP being genetically related to CCRCC [52]. Kuroda et al. also reported a loss of heterozygosity (LOH) in chromosome 3p in one MCRNLMP case [44]. Tretiakova et al. found a high rate of chromosome 3 abnormalities with chromosome 3 monosomy in 3/3 MCRNLMP cases [10]. Raspollini et al. conducted a comparison study between CCRCC and MCRNLMP using a genetic mutational analysis. There were no significant genetic differences between these two groups, except for *KRAS* mutation. According to their results, the *KRAS* mutation may be helpful for distinguishing between CCRCC and MCRNLP, despite their histologic similarities [53]. Kim et al. identified six novel genetic alterations, including *SET domain-containing 2* (*SETD2*), *lysine methyltransferase 2C* (*KMT2C*), *tuberous sclerosis complex 2* (*TSC2*), *GRB10 interacting GYF protein 2* (*GIGYF2*), *fibroblast growth factor receptor 3* (*FGFR3*) and *breakpoint cluster region protein* (*BCR*), also known as *renal carcinoma antigen NY-REN-26* (*BCR*), which could be potential candidate genes for differentiating between MCRNLMP and MCRCC [54].

## 6. Prognosis

The prognosis of MCRNLMP is excellent, with no cases of progression or metastatic spread [55]. This fact is based on multiple publications including more than 200 patients with clinical follow-ups longer than 5 years [1,5,6,9].

## 7. Discussion

Since the first report of MCRNLMP (then MCRCC) in 1982 [1], this entity has evolved, frequently being characterized, specified, named/re-named, and classified [2,3]. Firstly, it was characterized as a cystic neoplasm with less than 10% [2] and then less than 25% solid area [3]. Finally, MCRNLMP is described as a tumor entirely composed of cystic spaces with no expansive/solid nodules [56,57]. The original classification as multilocular cystic renal cell carcinoma (MCRCC) was re-designated as MCRNLMP, according to the ISUP recommendation, and became a part of the current WHO classification of renal tumors (2016) [5,12]. The nuclear grade (WHO/ISUP) of MCRNLMP is typically 1 (in two thirds of cases), or grade 2 (in one-third of MCRNLMP). WHO/ISUP grade 3 is not compatible with the diagnosis of MCRNLMP [10].

Chromosomal abnormalities were described in various studies, and chromosome 3p deletion was proved in 74% of MCRNLMP [52]. The *von Hippel-Lindau* (*VHL*) gene mutations were described in 25% of cases of MCRNLMP [47]. Furthermore, one case of loss of heterozygosity (LOH) in chromosome 3p in MCRNLMP was presented by Kuroda et al. [44].

The accurate incidence of MCRNLMP is not known, because of its rarity and variable diagnostic criteria used in various studies. However, it is estimated that MCRNLMP accounts for fewer than 1% of all renal neoplasms [16,23,58,59,60].

As with other cystic lesions of the kidney, MCRNLMP should be precisely diagnosed using proper imaging methods prior to treatment planning. The gold standard in imaging of the cystic tumors of the kidney is contrast-enhanced CT. The Bosniak classification is currently utilized to stratify the lesion accordingly [18,19,20,21,36]. In indeterminate cases where the initial CT imaging is not conclusive enough, a second imaging choice, such as MRI, needs to be utilized; some studies have demonstrated its benefit in diagnostics of cystic lesions of the kidney [24,25]. Other potential imaging modalities which can be used include contrast-enhanced ultrasound (CEUS) [26,27,28,29,61,62]. Typically, MCRNLMP is categorized as a cystic lesion, category Bosniak IIF or III [22,23]. Imaging studies cannot precisely distinguish MCRNLMP from other cystic lesions preoperatively [16,44,58,59,63].

The therapeutic management of MCRNLMP consists of strict clinical follow-ups or surgical interventions. There is still no strict protocol as to how and when to follow up Bosniak IIF category lesions. Weibl et al. suggested a CT scan in the follow-up every 6 months in the first 2 years, and then continuing with imaging studies once every year. The authors incorporated MRI into the follow-up regimen, which should be performed minimally in the first 4 years of follow-up [40]. In the past, Bosniak III lesions were strictly associated with surgical intervention. However, according to the recent EAU guidelines [42], it is possible to strictly follow-up such cases. The current preferred surgical approach is minimally invasive nephron-sparing surgery, which may allow the laparoscopic or robotic resection of such lesion, if technically feasible and oncological radicality is achievable.

In summary, MCRNLMP is a cystic lesion of the kidney with excellent prognosis. In 2016, the WHO separated this neoplasm of low malignant potential from cystic renal cell carcinomas, which have some overlapping morphologic features. Minimally invasive procedures (i.e., robotic surgery and laparoscopy) are preferred, with emphasis on nephron sparing surgery, if clinically feasible.

## Figures and Tables

**Table 1:** Differential diagnosis.

Entity in Differential Diagnosis	Clinical Characteristics	Imaging Studies	Macroscopic Findings	Microscopic Findings	Immunoprofile	Molecular Genetic Findings
**MCRNLMP**	Indolent behavior, frequently incidental finding, no clinical symptoms.	Mostly Bosniak III on CT/MRI	Variably large non-communicating cysts, no solid component	Cystic spaces lined by clear cells lining, low grade nuclei (WHO/ISUP grade 1–2), no expansile/solid nodular growth, no necrosis, no vascular invasion, no sarcomatoid changes	PAX8 +, CANH +, CK7 +, AMACR −, ER −, PR −	Chromosome 3p deletion, VHL mutation
**Renal** **cortical** **cyst**	Benign, symptoms only in big size lesion	Bosniak I or II on CT	Usually unilocular, thin-walled cortical cyst	Cystic space lined by single layer of cuboidal/flattened cells/atrophic epithelium	PAX8 −	No specific changes
**CCRCC with cystic changes (or regressive changes)**	Malignant lesion with favorable behavior compared with CCRCC	Bosniak III or IV on CT/MRI	Solid component, necrosis, hemorrhage may be present	Composed of cells with clear cytoplasm and distinct membrane, solid nodule present at least focally; necrosis, vascular invasion, and sarcomatoid changes may be present, even high-grade feature	PAX8 +, CANH +, CD10 +, AE1/3 +, Vimentin +, CK7 +/− (usually −/focally), AMACR −(usually), TFE3 −, HMB45 −, Melan A −	Chromosome 3p deletion, VHL mutation, VHL promoter methylation
**MEST**	Usually perimenopausal women, benign with possible rare malignant transformation	Bosniak III or IV on CT/MRI	Solitary, well circumscribed (unencapsulated), mixture of solid and cystic areas	Stromal (collagenous/edematous/spindle/ovarian-like) and epithelial (cysts of various size with flat/cuboidal/columnar/hobnail epithelial lining) component	PAX8 + (epithelium), ER + (stroma), PR + (stroma), inhibin + (stroma), HMB45 −, Melan A −	No specific changes
**MiT family RCC (some variant of Xp11 translocation RCC** [33]	Malignant, rare entity	Mostly Bosniak III or IV on CT/MRI	Multicystic mass, with a circumscribed appearance	Well-delimited, multilocular cystic lesion with thin membranous and fibrous septa, lined by a single layer of cell with clear to eosinophilic cytoplasm, WHO/ISUP grade 1/2 nuclei, no solid nodule	Cytokeratins +/−, TFE3 +, PAX8 +, CANH −	TFE3 gene rearrangements (MED15-TFE3 gene fusion)

MCRNLMP, multilocular cystic renal neoplasm of low malignant potential; CCRCC, clear cell renal cell carcinoma; MEST, mixed epithelial and stromal tumor; CANH, carbonic anhydrase; AMACR, alpha methyacyl CoA racemase; ER, estrogen receptors; PR, progesterone receptors; + positive; − negative; +/− variable.
